# The clinical value of long noncoding RNA GAS5 in acute ischemic stroke: Correlation with disease risk, inflammation, severity, and risk of recurrence

**DOI:** 10.1002/jcla.24171

**Published:** 2021-12-17

**Authors:** Pingping Fang, Yiping Wu, Zhongbo Zhang, Cui Cui, Xiaoxue Dong, Ke Hu, Jundong Jia, Xinfei Duan, Ying Zhang, Haoran Huo

**Affiliations:** ^1^ Department of Neurology Handan Central Hospital Handan China; ^2^ Department of Ophthalmology Handan Central Hospital Handan China; ^3^ Department of General Surgery Handan Central Hospital Handan China

**Keywords:** acute ischemic stroke, disease severity, inflammatory cytokines, LncRNA GAS5, recurrence

## Abstract

**Background:**

Long noncoding RNA growth arrest‐specific 5 (lnc‐GAS5) is involved in the pathophysiology of acute ischemic stroke (AIS) by regulating vascular stenosis, inflammation, and neurocyte apoptosis. This study aimed to explore the clinical value of lnc‐GAS5 in patients with AIS.

**Methods:**

Plasma samples were collected from 120 patients with AIS at admission and 60 controls after enrollment, and lnc‐GAS5 expression in the plasma of all participants was assessed by reverse transcription quantitative polymerase chain reaction. In patients with AIS, disease severity was evaluated using National Institute of Health Stroke Scale (NIHSS) score, and plasma inflammatory cytokine levels were measured by enzyme‐linked immunosorbent assay. Recurrence‐free survival (RFS) was calculated during a 36‐month follow‐up period.

**Results:**

Lnc‐GAS5 expression levels were higher in patients with AIS than in the controls (*p* < 0.001), and it had the potential to discriminate the controls from patients with AIS (area under the curve: 0.893, 95% confidence interval: 0.849–0.938). In patients with AIS, elevated lnc‐GAS5 levels were positively correlated with NIHSS score (*r* = 0.397, *p* < 0.001), diabetes mellitus (*p* = 0.046), and higher levels of tumor necrosis factor alpha (TNF‐α; *r* = 0.374, *p* < 0.001), interleukin‐6 (IL‐6; *r* = 0.223, *p* < 0.001), and interleukin‐17A (IL‐17A; *r* = 0.222, *p* = 0.015). The expression levels of lnc‐GAS5 were also negatively correlated with the levels of interleukin‐10 (IL‐10; *r* = −0.350, *p* < 0.001) and RFS (*p* = 0.036).

**Conclusion:**

Lnc‐GAS5 is correlated with higher susceptibility to AIS, inflammation, and severity, and can predict an increased risk of AIS recurrence, indicating that monitoring of lnc‐GAS5 might improve the management of AIS.

## Graphical abstract

Lnc‐GAS5 correlates with higher AIS susceptibility, inflammation and severity, and predicts increased AIS recurrence risk to some extent.

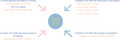

## 1 INTRODUCTION

Acute ischemic stroke (AIS) is a cerebrovascular disease induced by arterial stenosis or occlusion and is one of the leading causes of neurological morbidity and mortality worldwide.^1^, ^2^, ^3^ The risk factors for AIS include diabetes mellitus, carotid atherosclerosis, and dyslipidemia, among others.^1^, ^2^, ^3^, ^4^ The main treatment strategies for AIS include thrombolytic therapy, anticoagulation therapy, and neuroprotective therapy. However, patients with AIS still face long‐term adverse outcomes, such as cognitive and functional impairment.^5^, ^6^ Furthermore, until present, several studies have explored the potential biomarkers of AIS; however, recurrence risk and mortality of AIS are still elevating.^7^, ^8^, ^9^ Thus, reliable biomarkers to monitor disease risk, severity, and prognosis are crucial to improve the management of AIS.

Accumulating evidence suggests that long non‐coding RNA growth arrest‐specific 5 (lnc‐GAS5) regulates vascular stenosis, inflammation, and neurocyte apoptosis.^10^, ^11^, ^12^, ^13^, ^14^, ^15^ For instance, lnc‐GAS5 elevates the apoptosis of vascular endothelial cells, leading to vascular stenosis.^15^ Lnc‐GAS5 also aggravates inflammatory responses by targeting microRNA (miR)‐135a and modulating drosophila mothers against decapentaplegic protein 1 (Smad1).^11^, ^14^ In addition, various studies have shown that lnc‐GAS5 promotes neuronal cell apoptosis by modulating several microRNAs, such as miR‐137, miR‐21, miR‐26b‐5p, miR‐146a‐5p, and miR‐365a‐3p.^10^, ^11^, ^12^, ^13^, ^16^ Based on these data, we deduced that lnc‐GAS5 might play a vital role in the clinical management of AIS. However, most studies of lnc‐GAS5 in AIS have mainly focused on its role in the pathophysiology of AIS instead of its clinical value.^3^, ^17^, ^18^

Therefore, this study aimed to explore the correlation of lnc‐GAS5 with disease risk, disease severity, inflammation, and prognosis in AIS.

## 2 METHODS

### 2.1 Study participants

From February 2016 to December 2017, 120 first‐episode AIS patients treated in our hospital were consecutively recruited for this prospective study. Patients who met the following criteria were eligible for enrollment: (a) newly diagnosed with AIS according to the American Stroke Association guidelines^19^; (b) age >18 years; (c) with the occurrence of AIS within 12 h; (d) without intracranial hemorrhage; and (e) willing to be followed up regularly. The exclusion criteria were: (a) concomitant inflammatory disease or immune system disease; (b) presenting with active infections; (c) history of treatment with immunosuppressive agents within 3 months; (d) history of cancer or malignancy; and (e) pregnant or lactating. During the same period, 60 subjects who were at high risk of stroke were also enrolled as controls, and all of them had at least two of the following risk factors: current smoking, hypertension, hyperlipidemia, hyperuricemia, diabetes mellitus, and chronic kidney disease (CKD). The controls were also excluded from the study if they met any of the exclusion criteria for patients with AIS or had a history of stroke. Additionally, to eliminate age and sex as potential sources of bias, the age range of the controls was 55–80 years old, and the sex ratio was 3:2 (males to females). This study was approved by the institutional review board, and the signed informed consents were collected from all participants or their statutory guardians.

### 2.2 Data and sample collection

After enrollment, the clinical characteristics of the patients with AIS and controls were documented, including age, sex, body mass index (BMI), and risk factors for stroke. The National Institutes of Health Stroke Scale (NIHSS) scores of the patients with AIS were collected within 24 h after admission to assess disease severity. Peripheral blood (PB) samples were collected from patients with AIS on the day of admission and from the controls on the day of enrollment, and plasma was isolated for subsequent analyses.

### 2.3 Lnc‐GAS5 expression determination

The expression levels of lnc‐GAS5 in the plasma of all study participants were determined by reverse transcription quantitative polymerase chain reaction (RT‐qPCR). In brief, total RNA was extracted using the QIAamp RNA Blood Mini Kit (Qiagen) and reverse transcribed using the iScript™ cDNA Synthesis Kit (with random primers) (Bio‐Rad). qPCR was performed using SYBR^®^ Green Realtime PCR Master Mix (Toyobo). Relative lnc‐GAS5 expression was calculated using the $2^{‐\Delta \Delta C_{t}}$ method, and GAPDH was used as an internal reference. The primers used for qPCR were described in a previous study.^20^

### 2.4 Inflammatory cytokine determination

Enzyme‐linked immunosorbent assays (ELISAs) were performed to measure the plasma levels of inflammatory cytokines in patients with AIS, including tumor necrosis factor alpha (TNF‐α), interleukin‐6 (IL‐6), interleukin‐10 (IL‐10), and interleukin‐17A (IL‐17A). All ELISA kits were purchased from Bio‐Techne China (R&D Systems China Co., Ltd.) and were performed according to the manufacturer's protocol.

### 2.5 Assessment of recurrence‐free survival (RFS)

All AIS patients were followed up continuously until stroke recurrence, death, or completion of the 36‐month follow‐up. The final follow‐up date was December 29, 2020. RFS was calculated based on the follow‐up data. Patients who were lost to follow‐up for any reason were censored at the last visit.

### 2.6 Statistical analysis

Statistical analysis was performed using SPSS (version 22.0; IBM Corp.), and figures were generated using GraphPad Prism 7.02 software (GraphPad Software Inc.). Lnc‐GAS5 expression in patients with AIS was classified based on quantiles: quantile 1 (Q1), 0%–25%; quantile 2 (Q2), 26%–50%, quantile 3 (Q3), 51%–75%; and quantile 4 (Q4), 76%–100%. Comparisons between two groups were determined by Student's *t*‐test, Mann‐Whitney *U* test, and Chi‐square test. Receiver operating characteristic (ROC) curve analysis and the area under the curve (AUC) were used to estimate the ability of lnc‐GAS5 expression to discriminate patients with AIS from the controls. Correlations between two variables were analyzed using the linear‐by‐linear association test and Spearman's rank correlation test. The Kaplan–Meier method was used to determine RFS, and the correlation of lnc‐GAS5 expression with RFS was determined by the log‐rank test. Statistical significance was determined at a *P* value of less than 0.05.

## 3 RESULTS

### 3.1 Characteristics of patients with AIS and the controls

The mean age of the 120 patients with AIS was 67.7 ± 10.0 years; there were 35 (29.2%) women and 85 (70.8%) men. The mean age of the 60 controls was 66 ± 7.4 years; there were 24 (40%) women and 36 (60%) men. The demographic characteristics were not significantly different between the patients with AIS and the controls (*p* > 0.05 for all). The patients with AIS had an elevated prevalence of hyperuricemia (*p* = 0.011) and high‐risk factors (*p* = 0.009) compared to the controls. More detailed information is provided in Table 1.

**TABLE 1:** Characteristics of AIS patients and controls

Items	Controls (*N* = 60)	AIS patients (*N* = 120)	Statistic (*t*/*χ* ^2^/*Z*)	*p* value
Demographic characteristics				
Age (years), mean ± SD	66.0 ± 7.4	67.7 ± 10.0	−1.296	0.197
Gender, no. (%)			2.131	0.144
Female	24 (40.0)	35 (29.2)		
Male	36 (60.0)	85 (70.8)		
BMI (kg/m^2^), mean ± SD	23.9 ± 2.9	23.6 ± 2.2	0.815	0.416
Current smoke, no. (%)	29 (48.3)	51 (42.5)	0.551	0.458
Comorbidities and disease features				
Hypertension, no. (%)	44 (73.3)	100 (83.3)	2.500	0.114
Hyperlipidemia, no. (%)	29 (48.3)	56 (46.7)	0.045	0.833
Hyperuricemia, no. (%)	13 (21.7)	49 (40.8)	6.508	0.011
Diabetes mellitus, no. (%)	10 (16.7)	29 (24.2)	1.326	0.250
CKD, no. (%)	8 (13.3)	21 (17.5)	0.514	0.473
No. of risk factors			−2.631	0.009
1	0 (0.0)	6 (5.0)		
2	49 (81.7)	64 (53.3)		
3	9 (15.0)	32 (26.7)		
4	2 (3.3)	14 (11.7)		
5	0 (0.0)	4 (3.3)		
NIHSS score, median (IQR)	–	9.5 (7.0–12.0)	–	–
Inflammatory cytokines				
TNF‐α (pg/ml), median (IQR)	–	85.9 (64.1–115.2)	–	–
IL‐6 (pg/ml), median (IQR)	–	45.7 (35.4–62.4)	–	–
IL‐10 (pg/ml), median (IQR)	–	33.6 (24.4–52.4)	–	–
IL‐17A (pg/ml), median (IQR)	–	86.9 (64.6–128.2)	–	–

Abbreviations: AIS, acute ischemic stroke; BMI, body mass index; CKD, chronic kidney disease; IL‐10, interleukin‐10; IL‐17A, interleukin 17A; IL‐6, interleukin 6; IQR, interquartile range; NIHSS, National Institute Health of Stroke Scale; SD, standard deviation; TNF‐α, tumor necrosis factor alpha.

In the patients with AIS, the median NIHSS score was 9.5 (7.0–12.0). In terms of inflammatory cytokines, the median levels of TNF‐α, IL‐6, IL‐10, and IL‐17A were 85.9 (64.1–115.2) pg/ml, 45.7 (35.4–62.4) pg/ml, 33.6 (24.4–52.4) pg/ml, and 86.9 (64.6–128.2) pg/ml, respectively.

### 3.2 Comparison of lnc‐GAS5 levels between patients with AIS and controls

**FIGURE 1:**
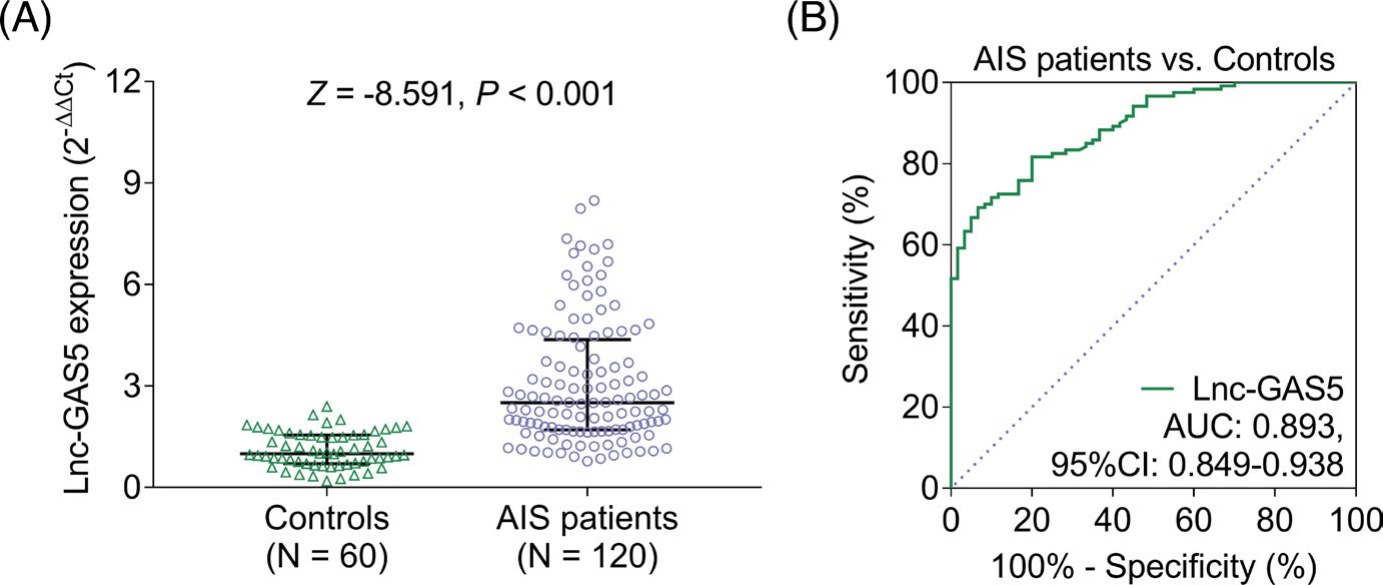
Lnc‐GAS5 levels in patients with AIS and controls. Comparison of lnc‐GAS5 expression levels between patients with AIS and controls (A); the ability of lnc‐GAS5 to differentiate patients with AIS from the controls (B). AIS, acute ischemic stroke; AUC, area under the curve; CI, confidence interval; lnc‐GAS5, long non‐coding RNA growth arrest‐specific 5

Lnc‐GAS5 levels were higher in patients with AIS (median [IQR]: 2.509 [1.707–4.369]) than in the controls (median [IQR]: 0.995 [0.707–1.555]; *p* < 0.001; Figure 1A). The ROC curve showed that lnc‐GAS5 could discriminate patients with AIS from the controls, with an AUC of 0.893 (95% confidence interval [CI] 0.849–0.938). The optimal cut off for lnc‐GAS5 expression was 1.865, with a sensitivity of 0.692 and specificity of 0.933 (Figure 1B).

### 3.3 Correlation of lnc‐GAS5 expression with NIHSS score and inflammatory cytokine levels in patients with AIS

**FIGURE 2:**
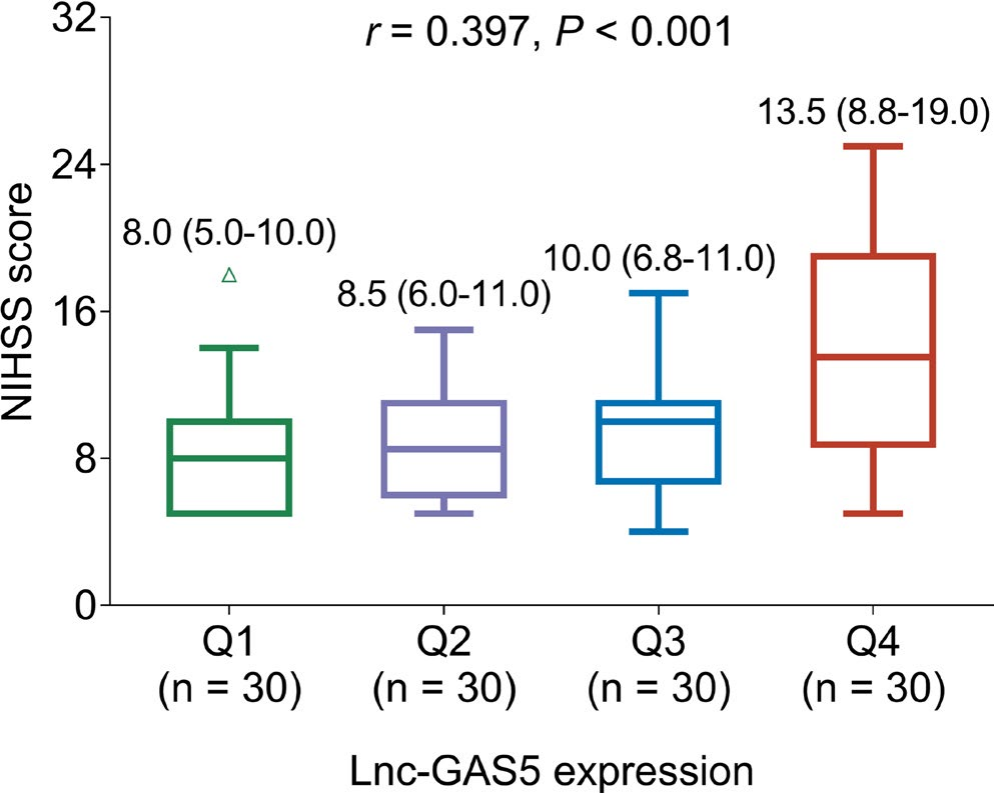
Correlation between lnc‐GAS5 expression level and NIHSS score. lnc‐GAS5, long non‐coding RNA growth arrest‐specific 5; NIHSS, National Institutes of Health Stroke Scale; Q, quantile

**FIGURE 3:**
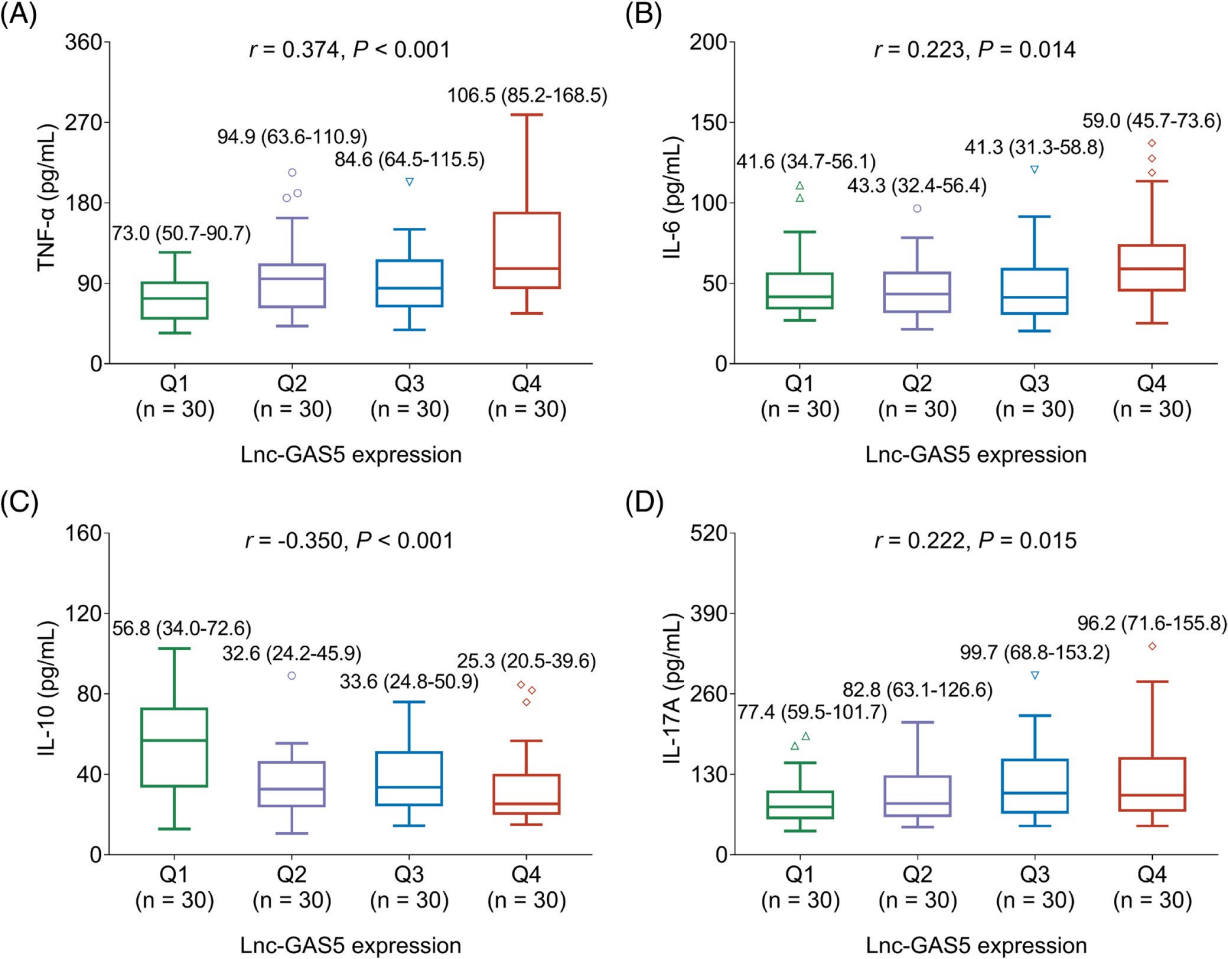
Correlation between the levels of lnc‐GAS5 and inflammatory cytokines. Correlation of lnc‐GAS5 expression levels with TNF‐α (A), IL‐6 (B), IL‐10 (C), and IL‐17A (D). IL‐10, interleukin‐10; IL‐17A interleukin‐17A; IL‐6, interleukin‐6; lnc‐GAS5, long non‐coding RNA growth arrest‐specific 5; Q, quantile; TNF‐α, tumor necrosis factor alpha

Increased lnc‐GAS5 expression was correlated with an elevated NIHSS score (*r* = 0.397, *p* < 0.001; Figure 2). Higher lnc‐GAS5 levels were correlated with increased TNF‐α (*r* = 0.374, *p* < 0.001; Figure 3A), IL‐6 (*r* = 0.223, *p* < 0.001; Figure 3B), and IL‐17A (*r* = 0.222, *p* = 0.015; Figure 3D) but decreased IL‐10 (*r* = −0.350, *p* < 0.001; Figure 3C). Elevated lnc‐GAS5 was also correlated with the prevalence of diabetes mellitus in patients with AIS (*p* = 0.046), but not with other comorbidities (Table 2).

**TABLE 2:** Correlation of lnc‐GAS5 expression with comorbidities in AIS patients

Items	Lnc‐GAS5 expression				Statistic (*χ^2^*)	*p* value
	Q1 (*n* = 30)	Q2 (*n* = 30)	Q3 (*n* = 30)	Q4 (*n* = 30)		
Hypertension, no. (%)						
No	6 (20.0)	6 (20.0)	5 (16.7)	3 (10.0)	1.190	0.275
Yes	24 (80.0)	24 (80.0)	25 (83.3)	27 (90.0)		
Hyperlipidemia, no. (%)						
No	16 (53.3)	23 (76.7)	12 (40.0)	13 (43.3)	2.656	0.103
Yes	14 (46.7)	7 (23.3)	18 (60.0)	17 (56.7)		
Hyperuricemia, no. (%)						
No	19 (63.3)	17 (56.7)	18 (60.0)	17 (56.7)	0.171	0.679
Yes	11 (36.7)	13 (43.3)	12 (40.0)	13 (43.3)		
Diabetes mellitus, no. (%)						
No	26 (86.7)	25 (83.3)	19 (63.3)	21 (70.0)	3.977	0.046
Yes	4 (13.3)	5 (16.7)	11 (36.7)	9 (30.0)		
CKD, no. (%)						
No	24 (80.0)	28 (93.3)	27 (90.0)	20 (66.7)	1.935	0.164
Yes	6 (20.0)	2 (6.7)	3 (10.0)	10 (33.3)		

Abbreviations: AIS, acute ischemic stroke; CKD, chronic kidney disease; Lnc‐GAS5, long non‐coding RNA growth arrest‐specific transcript 5; Q1, quartile 1.

### 3.4 Correlation of lnc‐GAS5 with RFS in patients with AIS

**FIGURE 4:**
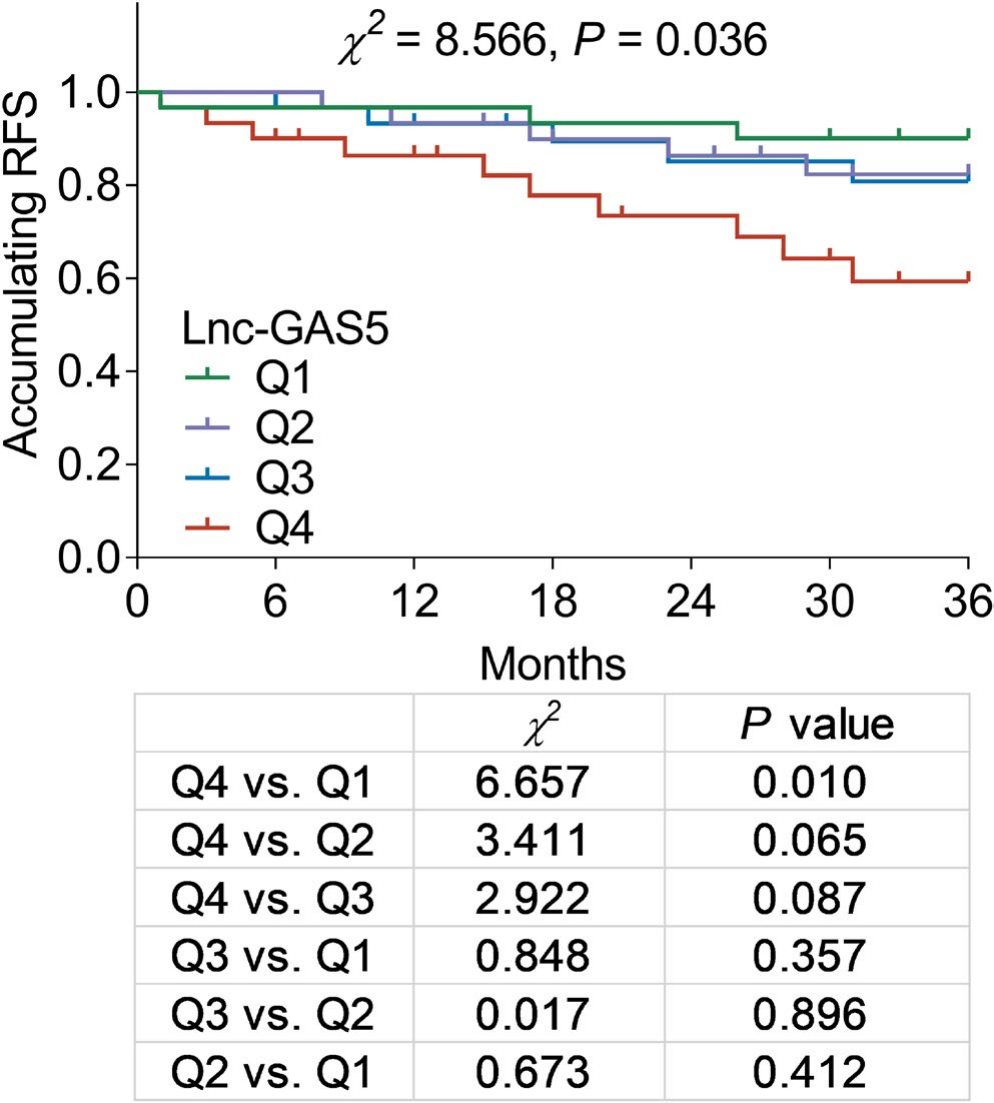
Correlation between lnc‐GAS5 levels and RFS. lnc‐GAS5, long non‐coding RNA growth arrest‐specific 5; Q1, quantile 1, 0.778–1.707; Q2, quantile 2, 1.707–2.509; Q3, quantile 3, 2.509–4.369; Q4, quantile 4, 4.369–8.484; RFS, recurrence‐free survival

To explore the correlation between lnc‐GAS5 and the prognosis of patients with AIS, patients with AIS were followed up for 36 months, which revealed that elevated lnc‐GAS5 was correlated with shorter RFS (*p* = 0.036), and RFS was significantly shorter in patients with lnc‐GAS5 levels in Q4 than in patients with lnc‐GAS5 levels in Q1 (*p* = 0.010; Figure 4).

## 4 DISCUSSION

In this study, we made several interesting observations: (1) lnc‐GAS5 expression was higher in patients with AIS than in the controls and was a good predictor of AIS risk; (2) lnc‐GAS5 was positively correlated with NIHSS score and pro‐inflammatory cytokine expression (TNF‐α, IL‐6, and IL‐17A), and was negatively correlated with anti‐inflammatory cytokine (IL‐10) in patients with AIS; and (3) lnc‐GAS5 was negatively associated with RFS in patients with AIS.

Regarding the role of lnc‐GAS5 in the risk of cardio‐cerebrovascular disease, a previous study showed that lnc‐GAS5 was obviously elevated in the plaques of atherosclerosis patients when compared to the levels in healthy populations.^21^ However, information on the correlation between lnc‐GAS5 and AIS risk is limited. Thus, we compared the expression of lnc‐GAS5 between AIS patients and healthy controls and used ROC curve analysis to estimate the predictive value of lnc‐GAS5 for AIS risk, which revealed that lnc‐GAS5 levels were higher in patients with AIS than in the controls, and lnc‐GAS5 had good predictive value for AIS risk. A possible explanation for this might be that: (1) increased lnc‐GAS5‐promoted apoptosis of vascular endothelial cells, resulting in arterial stenosis, which might play a role in the pathogenesis of AIS^15^, ^18^; (2) elevated lnc‐GAS5 could modulate enhancer of zeste homolog 2 (EZH2)‐mediated adenosine triphosphate‐binding cassette transporter A1 transcription to accelerate the progression of atherosclerosis, which might increase the risk of AIS^15^, ^22^; (3) lnc‐GAS5 might promote the formation of thrombosis, which could consequently accelerate AIS risk.^4^, ^23^ These data suggest that lnc‐GAS5 might be useful as a novel biomarker of AIS risk. We also found that elevated lnc‐GAS5 was correlated with the prevalence of diabetes mellitus in patients with AIS, suggesting that an increase in lnc‐GAS5 might promoted the progression of diabetes mellitus,^24^consequently, diabetes could accelerate vascular aging, which might lead to the development of AIS.^25^

The correlation of lnc‐GAS5 with disease severity or inflammation in AIS has been rarely reported; thus, to explore this issue, for patients with AIS, the NIHSS score were collected to evaluate disease severity, and the levels of some inflammatory cytokines were determined. The results showed that lnc‐GAS5 was positively correlated with disease severity and inflammation. The potential reasons for this might be that: (1) lnc‐GAS5 might accelerate inflammatory responses by regulating Smad1 in AIS, which could lead to increased inflammation^11^; (2) lnc‐GAS5 could promote vascular intima thickening by regulating the apoptosis of macrophages and vascular endothelial cells, which might aggravate atherosclerotic lesions and consequentially increase the severity of AIS^15^; (3) lnc‐GAS5 might induce neurological impairment by regulating neuronal apoptosis and aggravating cerebral ischemic damage *via* Notch1 signaling pathway, which might lead to cognitive and functional impairment, consequently increasing the severity of AIS^10^, ^11^, ^12^, ^13^; (4) lnc‐GAS5 might accelerate thrombogenesis, which could lead to higher severity of AIS.^23^ These findings suggest that lnc‐GAS5 may reflect disease severity and inflammation in AIS.

Recurrence is still a serious challenge in AIS, as it can result in prolonged hospitalization, unfavorable functional and cognitive outcomes, and increased mortality.^26^ Thus, the identification of a reliable biomarker to predict the recurrence of AIS is crucial. Hence, this study investigated the correlation between lnc‐GAS5 and recurrence of AIS, which revealed that elevated lnc‐GAS5 was associated with decreased RFS in AIS patients. The potential reason for this association might be that increased levels of lnc‐GAS5 could accelerate disease progression and increase disease severity by upregulating inflammation and neuronal apoptosis and promote vascular intima thickening, which might decrease RFS.^11^, ^12^, ^15^, ^16^ Thus, lnc‐GAS5 is correlated with an unfavorable prognosis in AIS.

This study has some limitations: (1) the follow‐up duration was relatively short, and the correlation between lnc‐GAS5 and the long‐term prognosis of AIS patients could be investigated in a future study; (2) the mechanism by which lnc‐GAS5 through its targets is involved in the pathogenesis and progression of AIS was not investigated, and thus could be explored in future studies; (3) because this study was a single‐center study and the enrolled patients might have been from neighboring regions, there might have been patient selection bias; (4) the sample size of this study was relatively small, larger sample size could be analyzed in the further study; (5) the disease controls such as other neurological diseases apart from stroke, including Parkinson and Alzheimer disease, could be considered in the further study.

## 5 CONCLUSION

In conclusion, lnc‐GAS5 is correlated with higher susceptibility to AIS and greater inflammation and severity and could predict the risk of recurrence to some extent, indicating that monitoring of lnc‐GAS5 might improve the management of AIS.

## CONFLICT OF INTEREST

The authors declare that no conflict of interest.

## Data Availability

The datasets used and/or analyzed during this study are available from the corresponding author on reasonable request.
